# Disruption of Smad7 Promotes ANG II-Mediated Renal Inflammation and Fibrosis via Sp1-TGF-β/Smad3-NF.κB-Dependent Mechanisms in Mice

**DOI:** 10.1371/journal.pone.0053573

**Published:** 2013-01-03

**Authors:** Guan-Xian Liu, You-Qi Li, Xiao R. Huang, Lihua Wei, Hai-Yong Chen, Yong-Jun Shi, Rainer L. Heuchel, Hui Y. Lan

**Affiliations:** 1 Department of Nephrology, Central Municipal Hospital of Huizhou, Guangdong, China; 2 CUHK Research Institute, Shenzhen, Guangdong, China; 3 Department of Medicine and Therapeutics, and Li Ka Shing Institute of Health Sciences, The Chinese University of Hong Kong, Hong Kong SAR, China; 4 CLINTEC, Karolinska Institutet, Stockholm, Sweden; New York Medical College, United States of America

## Abstract

Smad7 is an inhibitory Smad and plays a protective role in obstructive and diabetic kidney disease. However, the role and mechanisms of Smad7 in hypertensive nephropathy remains unexplored. Thus, the aim of this study was to investigate the role and regulatory mechanisms of Smad7 in ANG II-induced hypertensive nephropathy. Smad7 gene knockout (KO) and wild-type (WT) mice received a subcutaneous infusion of ANG II or control saline for 4 weeks via osmotic mini-pumps. ANG II infusion produced equivalent hypertension in Smad7 KO and WT mice; however, Smad7 KO mice exhibited more severe renal functional injury as shown by increased proteinuria and reduced renal function (both p<0.05) when compared with Smad7 WT mice. Enhanced renal injury in Smad7 KO mice was associated with more progressive renal fibrosis with elevated TGF-β/Smad3 signalling. Smad7 KO mice also showed more profound renal inflammation including increased macrophage infiltration, enhanced IL-1β and TNF-α expression, and a marked activation of NF-κB signaling (all p<0.01). Further studies revealed that enhanced ANG II-mediated renal inflammation and fibrosis in Smad7 KO mice were also associated with up-regulation of Sp1 but downregulation of miR-29b expression. Taken together, the present study revealed that enhanced Sp1-TGF-β1/Smad3-NF-κB signaling and loss of miR-29 may be mechanisms by which deletion of Smad7 promotes ANG II-mediated renal fibrosis and inflammation. Thus, Smad7 may play a protective role in ANG II-induced hypertensive kidney disease.

## Introduction

Hypertensive nephropathy, which is characterized by progressive renal fibrosis and inflammation, is a major complication of hypertension and is one of the main causes of chronic kidney disease [1]. It is widely accepted that angiotensin II (ANG II) is a key mediator in hypertensive nephropathy and plays an essential role in the progression of chronic kidney disease [2], [3]. This is supported by the finding that blockade of ANG II actions with ACE inhibitors or angiotensin type 1 (AT1) receptor antagonists can inhibit disease progression in human and experimental kidney disease [4], [5]. It is now clear that ANG II can activate several intracellular signaling pathways to mediate renal fibrosis and inflammation, including TGF-β/Smads, nuclear factor-kappa B (NF-κB), and mitogen-activated protein kinases (MAPK) [6]–[10]. However, how these pathways are integrated in ANG II-mediated hypertensive nephropathy remains largely unclear.

In the context of renal fibrosis, ANG II induces extracellular matrix production through TGF-β-dependent and independent mechanisms [9], [10]. Within the TGF-β/Smad signaling cascade, Smad3, but not Smad2, is a critical downstream mediator responsible for renal and cardiovascular fibrosis [10]–[15]. Indeed, many genes involved in tissue fibrosis (e.g. ColIa1, ColIa2, ColIIIa1, ColVa2, ColVIa1, ColVIa3 and tissue inhibitor of matrix metalloproteinase-1) are regulated by TGF-β/Smad3 signaling [16]. Thus, Smad3 plays an essential role in ANG II-mediated fibrosis in vivo and in vitro [10]–[15].

Smad7 is an inhibitory Smad that negatively regulates TGF-β/Smad-mediated renal fibrosis by facilitating degradation of TGF-β receptor-1 and Smads via the Smurf2 and arkadia-dependent ubiquitin-proteasome mechanism [17]–[20]. In chronic kidney diseases, many mediators such as TGF-β1 and ANG II are able to induce Smad7 mRNA expression, but Smad7 protein is degraded [11], [19]–[23]. As an adaptor protein for E3 ubiquitin ligases such as Smurf2 and arkadia [17], [20], Smad7 is degraded once this ubiquitin cascade becomes activated. In renal fibrosis, Smad7 protein levels are reduced. Evidence for protective role of Smad7 in renal fibrosis comes from studies in which Smad7 gene knockout (KO) mice develop worse fibrosis in obstructed nephropathy [23], [24]. It has been reported that renal Smad7 levels are reduced in the rat remnant kidney and in cells in response to ANG II [11], [21]. However, the role of Smad7 in hypertensive nephropathy in response to ANG II remains unexplored. Therefore, in this study we determined the function and underlying mechanisms of Smad7 in ANG II-mediated hypertensive nephropathy through the use of Smad7 KO mice.

## Materials and Methods

### A Mouse Model of ANG II-induced Hypertension

Smad7 KO mice are generated in a CD-1 background mouse strain from which functional Smad7 is disrupted by deleting exon I in the Smad7 gene as previously described [25]. It is reported that the CD-1 stain is more susceptible to renal injury in response to ANG II [26]. Therefore, a mouse model of hypertensive nephropathy was induced in littermate Smad7 KO or WT mice (male mice, aged 8 weeks, 20–25 g) by subcutaneous infusion of ANG II at a dose of 1000 ng/kg/min for 28 days via osmotic minipumps as described previously [14], [15]. Blood pressure was measured weekly by the tail-cuff method using the CODA noninvasive blood pressure system (Kent Scientific, Torrington, CT) in conscious mice according to the manufacturer's instructions. Kidney tissue samples were collected at day 28 for histology, immunohistochemistry, Western blot, and real-time PCR analyses as described previously [13]–[15]. Control mice received a saline infusion instead of ANG II, following the same protocol. The experimental procedures were approved by the Animal Experimental Committee of The Chinese University of Hong Kong (Permit No. 1165-05).

### Proteinuria and Renal Function Analysis

Twenty-four hour urine samples were collected before and weekly during ANG II infusion. Urine protein levels were measured using the Quick start Bradford Dye Reagent (Bio-RAD). Levels of both serum creatinine and urinary creatinine were detected by the Enzymatic creatinine LiquiColor Reagent (Stanbio Laboratory, Boerne, TX), according to the manufacturer’s instructions.

### Histology and Immunohistochemistry

Renal morphology was examined in methyl Carnoy’s-fixed, paraffin-embedded tissue sections (4 µm) stained with periodic acid-Schiff (PAS) reagent. Immunohistochemistry was performed in paraffin sections using a microwave-based antigen retrieval method [22]. Primary antibodies used in the present study were: collagen I (Southern Technology, Birmingham, AL), α-SMA (Sigma, St. Louis, MO), TNFα, IL-1β, TGF-β1, phospho-Smad2/3 (Santa Cruz Biotechnology, Santa Cruz, CA), phospho-NFκB/p65 (Cell Signaling Technology, Danvers, MA), CD3 (Abcam, Cambridge, MA), and F4/80 (Serotec, Oxford, UK). After being incubated with the secondary antibody, sections were developed with diaminobenzidine to produce a brown product and counterstained with hematoxylin. The percentage of positive area was measured using a quantitative image-analysis system (Image-Pro Plus 6.5, Media Cybernetics, Silver Spring, MD) as previously described [13], [14]. Briefly, the area of glomeruli and the tubulointerstitium was outlined, and the positive signal measured and expressed as the percentage of the area examined. For quantitative analysis of F4/80^+^ macrophages, phospho-Smad3, and phospho-p65^+^ cells, 20 consecutive glomeruli were counted and data expressed as cells/glomerular cross-section (gcs), whereas positive cells in the tubulointerstitium were counted under high-power fields (×40) by means of a 0.25-mm^2^ graticule fitted in the eyepiece of the microscope and expressed as cells per millimeter squared.

### Western Blot Analysis

Protein from kidney tissues was extracted with RIPA lysis buffer for Western blot analysis as previously described [13]–[15]. Nitrocellulose membranes were probed overnight with primary antibodies against phospho-p65 (ser276), phospho-IκBα (ser32), IκBα, phospho-Smad3 (s423–425) (Cell Signaling Technology), Sp1 (H-225), p65, IκBα, Smad7 (Santa Cruz Biotechnology), Smad3 (Zymed, San Francisco, CA), collagen I, α-SMA, or GAPDH (Chemicon, Temecula, CA, USA), followed by incubation with LI-COR IRDye 800-labeled secondary antibodies (Rockland Immunochemicals, Gilbertsville, PA). The signal was detected with Odyssey Infrared Imaging System (Li-COR Biosciences, Lincoln, NE) and quantified using the Image J program (National Institutes of Health). Protein levels are expressed relative to the GAPDH control and presented as mean ± SE.

### Real-time PCR

Total kidney RNA was isolated using the RNeasy kit, according to the manufacturer’s instructions (Qiagen, Valencia, CA) and mRNA levels of collagen I, α-SMA, TGF-β1, IL-1β, and TNF-α were measured by real-time PCR with primers as previously described [13]–[15]. Sp1 was detected with primers: forward 5′-GCTGCCACCATGAGCGACCAA- 3′, reverse 5′-CACCGCCACCATTGCCGCTA- 3′. For detection of miR-29b expression, renal RNA was isolated using Trizol® and expression of miR-29b was examined with primers as previously described [27]. The housekeeping gene GAPDH or U6 was used as an internal control. Expression of the gene of interest relative to the internal control is presented as mean ± standard error (SE).

### Statistical Analysis

Data are expressed as mean ± SE. Statistical analysis was performed using one-way analysis of variance (ANOVA), followed by Newman-Keuls Post Test from the Prism Program (Prism 5.0 GraphPad Software, San Diego, CA).

## Results

### Deletion of Smad7 Enhances ANG II-mediated Hypertensive Nephropathy

**Figure 1:**
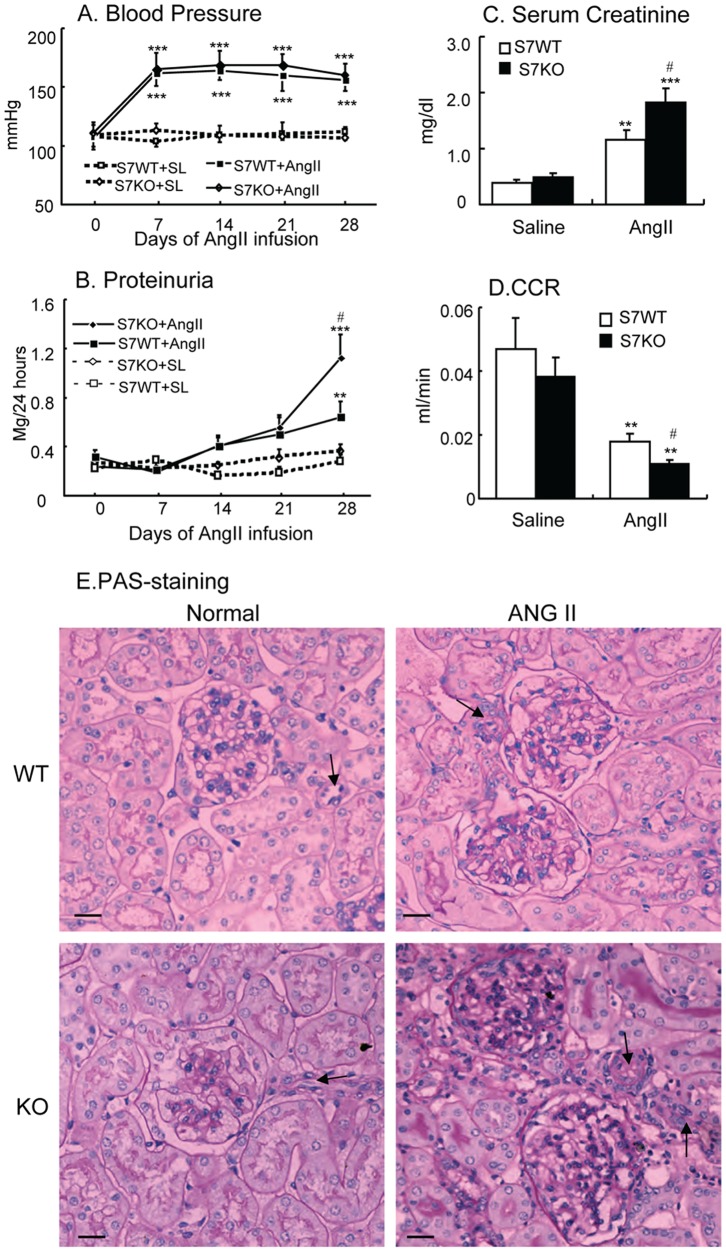
Deletion of Smad7 enhances ANG II-induced renal injury. **A:** Systolic blood pressure. **B:** Proteinuria. **C: S**erum creatinine. **D:** Creatinine clearance (CCR). **E:** Histological damage [periodic acid-Schiff (PAS)-stained sections]. Note that disruption of Smad7 enhances ANG II-mediated renal injury, including higher levels of proteinuria and serum creatinine, a greater fall in CCR, and histological damage such as glomerular hypercellularity, vascular sclerosis (arrows), and ECM deposition when compared with Smad7 WT mice, despite equal levels of high blood pressure. Values are means ± SE for groups of 6 mice. Scale bar, 50 µM. *P<0.05, **P<0.01, ***P<0.001 compared with saline (SL) control mice.^ #^ *P*<0.05,^ ##^ *P*<0.01, ^###^ *P*<0.001 compared with ANG II-infused Smad7 WT mice.

After Ang-II infusion, both WT and Smad7 KO mice developed an equivalent increase in blood pressure over days 7 to 28 (Fig. 1A). However, Smad7 KO mice had higher levels of proteinuria, serum creatinine, and a greater fall in the rate of creatinine clearance (CCR) when compared to WT mice (Fig. 1, B–D). PAS staining of kidney tissue showed that chronic ANG II infusion caused more severe glomerular and vascular hypercellularity and increased extracellular matrix accumulation within the mesangium and tubulointerstitium in Smad7 KO mice when compared to WT mice (Fig. 1E).

### Deletion of Smad7 Promotes Renal Fibrosis and Inflammation in ANG II-induced Hypertension

**Figure 2:**
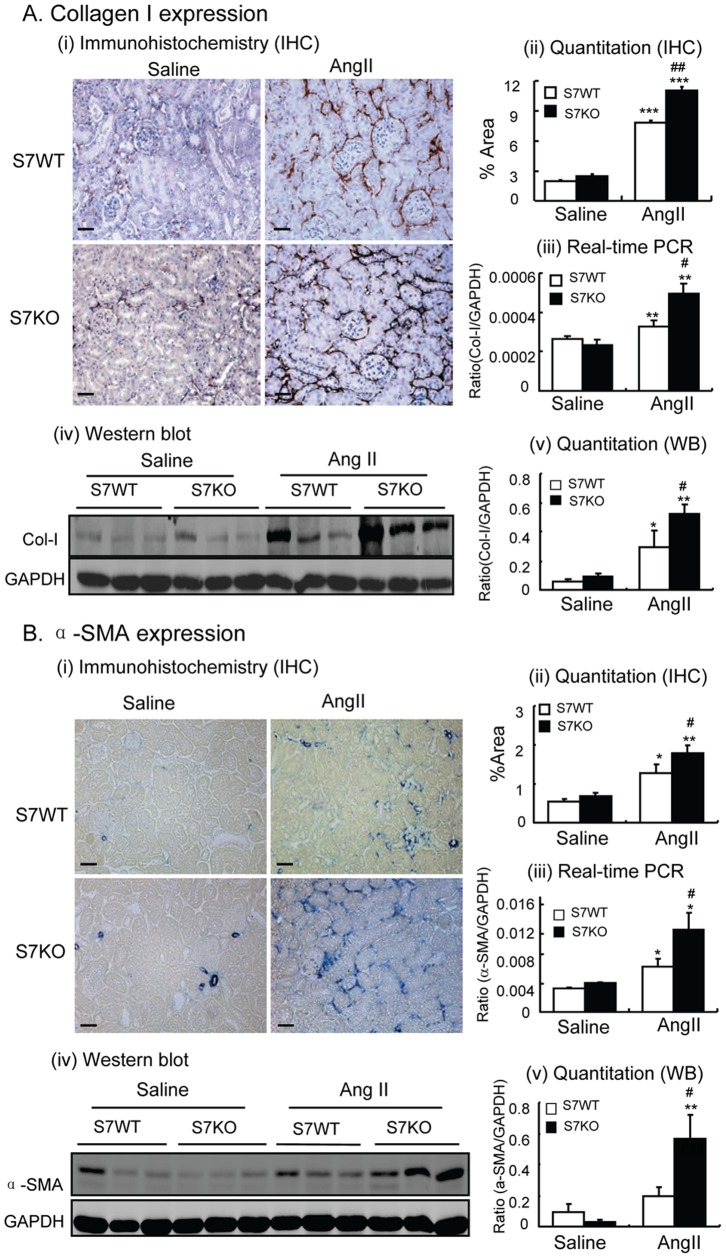
Deletion of Smad7 enhances ANG II-induced renal fibrosis. **A:** Collagen I. **B:** α-SMA. Immunohistochemistry (IHC, i, ii), real-time PCR (iii), and Western blot (WB, iv, vi) analyses show that deletion of Smad7 enhances ANG II-induced renal fibrosis when compared with Smad7 WT mice. Each bar represents means ± SE for groups of 6 mice. Scale bar, 50 µM. *P<0.05, **P<0.01, ***P<0.001 compared with saline (SL) control mice. ^#^ *P*<0.05, ^##^ *P*<0.01, ^###^ *P*<0.001 compared with ANG II-infused Smad7 WT mice.

Immunohistochemistry revealed that compared with the saline-treated mice, chronic ANG II infusion resulted in renal fibrosis in WT mice, as demonstrated by a significant increase in collagen I and α-SMA expression in both mRNA and protein levels, and the accumulation of α-SMA^+^ myofibroblasts in the tubulointerstitium (Fig. 2). These fibrotic responses were significantly enhanced in Smad7 KO mice with chronic ANG II infusion as shown in Figure 2.

**Figure 3:**
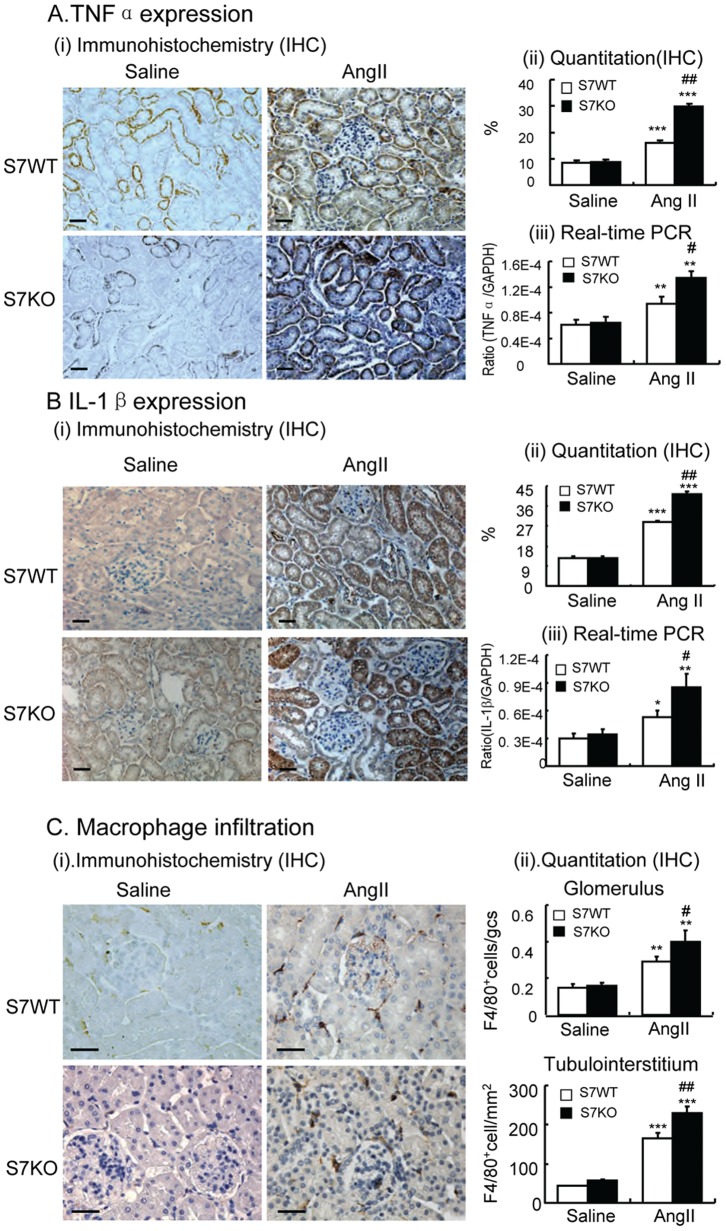
Deletion of Smad7 enhances ANG II-induced renal inflammation. **A:** TNFα. **B:** IL-1β. **C:** F4/80^+^ macrophages. Immunohistochemistry (IHC, i, ii) and real-time PCR (iii) analyses show that deletion of Smad7 enhances ANG II-induced renal inflammation with up-regulation of TNFα and IL-1β, and an increased macrophage infiltrate when compared with Smad7 WT mice. Each bar represents means ± SE for groups of 6 mice. Scale bar, 50 µM. *P<0.05, **P<0.01, ***P<0.001 compared with saline (SL) control mice. ^#^ *P*<0.05, ^##^ *P*<0.01 when compared with ANG II-infused Smad7 WT mice.

ANG II infusion also induced inflammation in the kidney of WT mice as shown by increasing mRNA and protein levels of the pro-inflammatory cytokines TNF-α and IL-1β, as well as by a significant macrophage infiltrate (Fig. 3). Again, ANG II-induced these inflammatory responses were largely enhanced in Smad7 KO mice (Fig. 3).

### Enhanced Activation of TGF-β1/Smad and NF-κB Signaling Contributes to ANG-II Mediated Renal Fibrosis and Inflammation in Smad7 KO Mice

**Figure 4:**
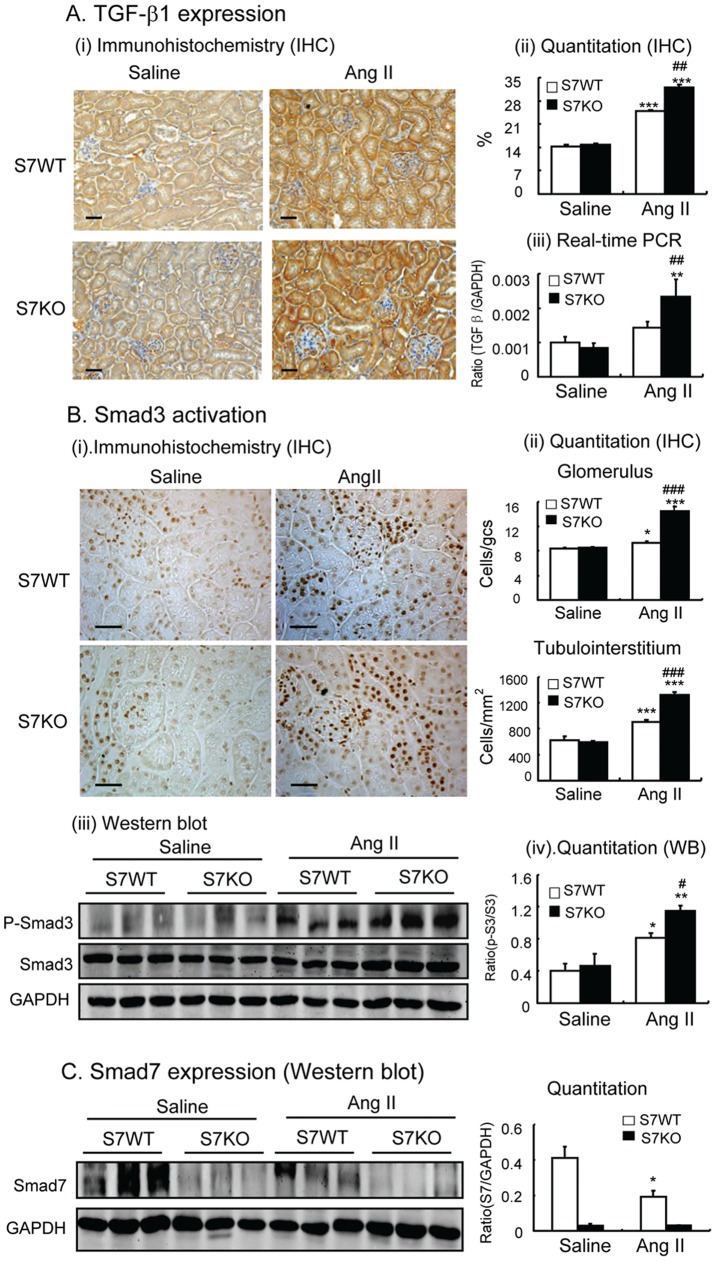
Deletion of Smad7 enhances ANG II-induced activation of TGF-β1/Smad3 signaling in the kidney. **A:** TGF-β1 expression detected by immunohistochemistry (i ii) and real-time PCR (iii). **B:** Activation of Smad3 determined by immunohistochemistry for phospho-Smad2/3 nuclear translocation (i, ii) and Western blots for phosphorylation levels of Smad3 in the kidney (iii, iv). **C:** Smad7 protein levels. Note that ANG II induces degradation of renal Smad7 protein in the Smad7 WT. Each bar represents means ± SE for groups of 6 mice. Scale bar, 50 µM. *P<0.05, **P<0.01, ***P<0.001 compared with saline (SL) control mice. ^#^ *P*<0.05, ^##^ *P*<0.01,^ ###^ *P*<0.001 when compared with ANG II-infused Smad7 WT mice.

**Figure 5:**
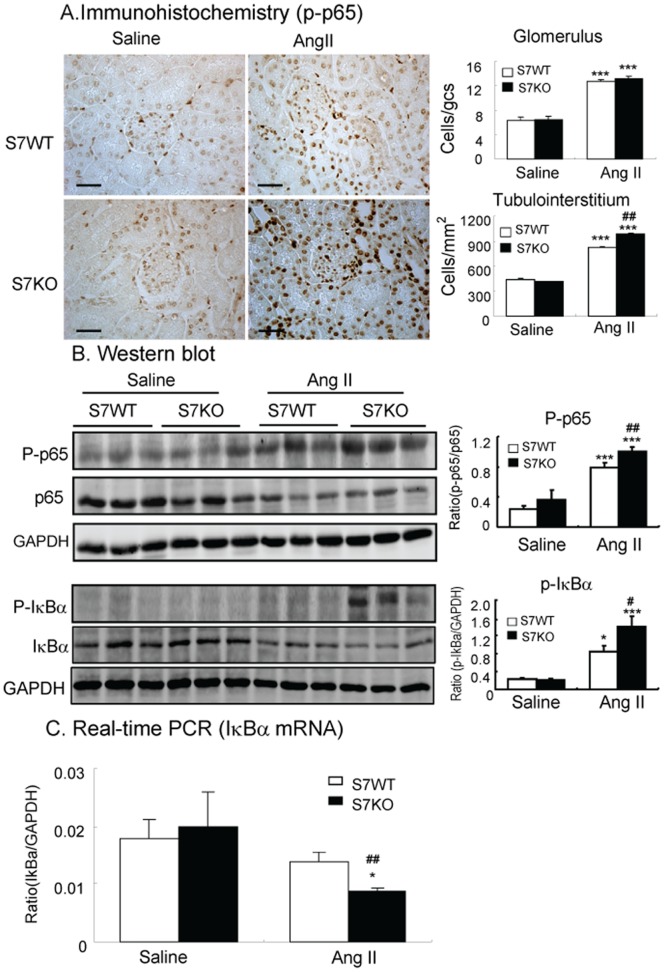
Deletion of Smad7 enhances ANG II-induced activation of NF-κB/p65 in the kidney. **A:** Immunohistochemistry detects that deletion of Smad7 enhances phospho-NF-κB/p65 nuclear translocation in the hypertensive kidney. **B:** Western blot analysis shows that disruption of Smad7 promotes IκBα degradation through phosphorylation, thereby enhancing NF-κB activation as determined by significantly increasing phosphorylation of p65 in the hypertensive kidney. **C.** Real-time PCR shows that deletion of Smad7 significantly inhibits IκBα mRNA expression in mice after ANG II infusion. Each bar represents means ± SE for groups of 6 mice. Scale bar, 50 µM. *P<0.05, ***P<0.001 compared with saline (SL) control mice. ^#^ *P*<0.05,^ ##^ *P*<0.01 when compared with ANG II-infused Smad7 WT mice.

To investigate the mechanisms by which deletion of Smad7 promoted Ang-II induced renal fibrosis and inflammation, we studied TGF-β/Smad and NF-κB signaling pathways in the hypertensive kidney. Immunohistochemistry, real-time PCR and Western blot analysis revealed that ANG II infusion significantly up-regulated renal TGF-β1 expression (Fig. 4A), resulting in high levels of phospho-Smad2/3 and its nuclear translocation (Fig. 4B), and a reduction of Smad7 protein levels in WT mice (Fig. 4C). ANG II-induced upregulation of TGF-β1 and activation of Smad signaling was further enhanced in Smad7 KO mice (Fig. 4). Furthermore, both immunohistochemistry and Western blot analyses revealed that although ANG II infusion caused significant activation of the NF-κB signaling pathway, disruption of Smad7 resulted in enhanced activation of NF-κB/p65 in terms of increased NF-κB/p65 phosphorylation and nuclear translocation (Fig. 5A and B), which was associated with a reduction in the NF-κB inhibitor, IκBα, on the basis of increased IκBα degradation by phosphorylation while decreasing its mRNA expression (Fig. 5B and C).

### Deletion of Smad7 Upregulates Sp1 but Downregulates miR-29b in ANG II-mediated Hypertensive Nephropathy

**Figure 6:**
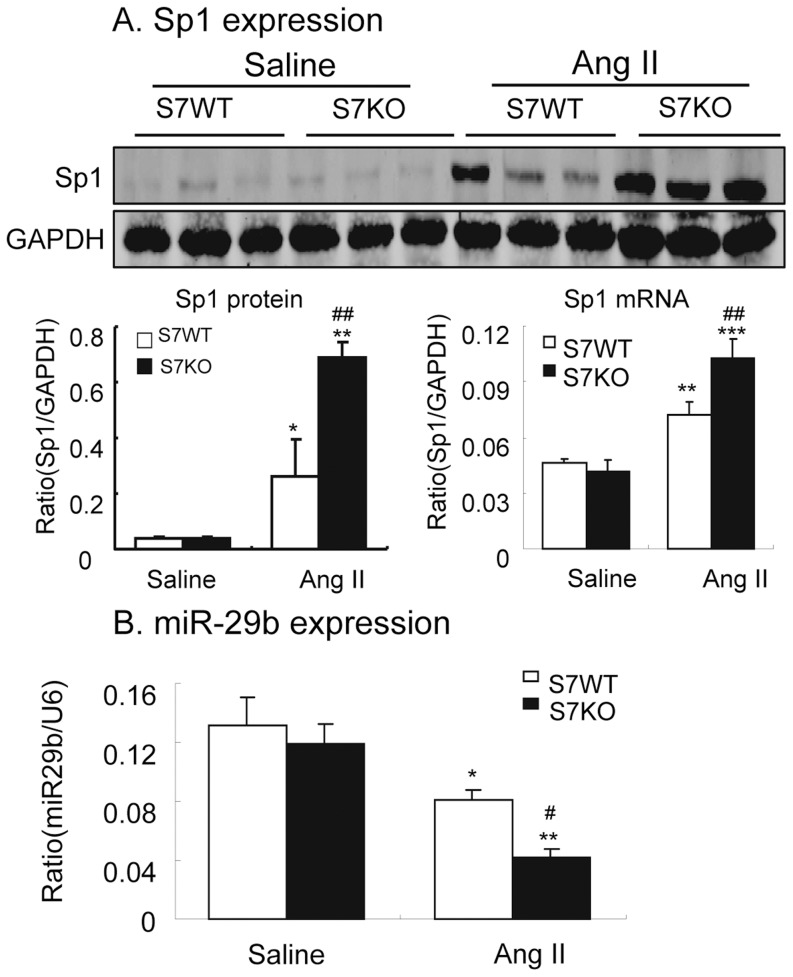
Deletion of Smad7 enhances upregulation of Sp1 but dowregulates miR-29b expression in ANG II-induced hypertensive nephropathy. **A.** Western blot and real-time PCR analysis show that disruption of Smad7 enhances ANG II-induced upregulation of Sp1 at both protein and mRNA levels. **B.** Real-time PCR detects that disruption of Smad7 results in a further inhibition of miR-29b expression in the hypertensive kidney. Each bar represents means ± SE for groups of 6 mice. *P<0.05, **P<0.01, ***P<0.001 compared with saline (SL) control mice. ^#^ *P*<0.05, ^##^ *P*<0.01 when compared with ANG II-infused Smad7 WT mice.

The transcriptional factor Sp1 has been shown to mediate ANG II activities and can interact with both TGF-β/Smad and NF-κB pathways [28]–[32]. We thus examined expression of Sp1 in ANG II-mediated kidney disease. As shown in Figure 6 (A), renal Sp1 expression at the mRNA and protein level was up-regulated by ANG II infusion in WT mice, which was further enhanced in the hypertensive kidney of Smad7 KO mice.

We have recently shown that miR-29b is a downstream target of TGF-β/Smad3 signalling and that down-regulation of miR-29b promotes renal fibrosis [27]. As miR-29b is also negatively regulated by the NF-κB-YY1-miR-29 regulatory circuit [33], we hypothesized that a loss of renal miR-29b might contribute to the enhanced renal fibrosis and inflammation seen in ANG II infused Smad7 KO mice. As shown in Figure 6B, renal levels of miR-29b were significantly reduced in ANG II infused WT mice and this was further decreased in Smad7 KO mice in response to ANG II.

## Discussion

Although it has been reported that deletion of Smad7 enhances renal inflammation and fibrosis in obstructive nephropathy and diabetic nephropathy [24], [34], role of Smad7 in ANG II-mediated hypertensive nephropathy, a major cause of end-stage kidney disease, remains unexplored. The present study identified that Smad7 plays a protective role in ANG II-mediated hypertensive nephropathy. The findings that disruption of Smad7 enhanced ANG II-induced renal injury by promoting TGF-β/Smad2/3-mediated renal fibrosis and NF-κB-driven renal inflammation added new information for a better understanding of the mechanism of Smad7 in negatively regulating ANG II-mediated kidney injury. Furthermore, the findings that enhanced TGF-β/Smad3-mediated renal fibrosis and NF-κB-dependent renal inflammation in Smad7 KO mice were associated with up-regulation of Sp1 and down-regulation of miR-29b in the hypertensive kidney also provided new mechanisms for understanding the protective role of Smad7 in ANG II-mediated hypertensive nephropathy. Thus, we identified that ANG II-mediated hypertensive kidney injury involves the complex and integrated mechanisms. Of these, Smad7 is an important regulator which operates via direct and indirect mechanism to suppress ANG II-mediated renal fibrosis and inflammation.

An important finding in the present study is that loss of Smad7 promoted ANG II-induced activation of TGF-β/Smad signaling, resulting in a progressive renal fibrosis in a mouse model of hypertension. This finding *largely extended* a previous observation *in vitro* that ANG II-induced TGF-β/Smad3-mediated renal fibrosis in cultured renal tubular epithelial cells is associated with down-regulation of Smad7 via AT1R-Smurf2-dependent ubiquitin degradation of Smad7 [11]. It has been shown that after binding to the AT1 receptor, ANG II induces fibrosis by directly activating TGF-β/Smad signaling and by indirectly activating TGF-β/Smad signaling via a ERK/p38 MAP kinase-Smad cross-talk mechanism [9], [10]. We recently demonstrated that Smad3, but not Smad2, is the key transcription factor responsible for TGF-β-mediated renal fibrosis [10]–[13]. Thus, deletion of Smad3 is capable of preventing ANG II-mediated cardiac remodeling and renal fibrosis [14], [15]. The present finding that disrupted Smad7 enhanced Smad3-mediated renal fibrosis added a new evidence for a protective role of Smad7 in ANG II-mediated hypertensive nephropathy.

Enhanced NF-κB-driven renal inflammation may be the key mechanism through which disruption of Smad7 promoted ANG II-induced renal inflammation as demonstrated by up-regulation of pro-inflammatory cytokines (IL-1β and TNF-α) and infiltration of F4/80^+^ macrophages. This is consistent with the previous findings in non-hypertensive kidney disease in which disruption of Smad7 promots NF-κB-dependent renal inflammation in obstructive and diabetic nephropathy [24], [34], arguing that Smad7 is of general importance in the regulation of renal inflammation under various pathological conditions. It has been reported that overexpression of Smad7 is capable of inhibiting NF-κB-driven renal inflammation by up-regulating the NF-κB inhibitor, IκBα [34]–[37]. By using Smad7 KO mice, the present study provided new evidence that inhibition of IκBα mRNA expression and promoted its degradation by phosphorylation may be an essential mechanism by which deletion of Smad7 enhanced ANG II-induced activation of NF-κB/p65 and renal inflammation.

Interestingly, we also found that deletion of Smad7 promoted ANG II-induced upregulation of Sp1 in the hypertensive kidney. This new finding suggests that enhanced ANG II-induced Sp1 pathway may be an additional mechanism by which mice lacking Smad7 were promoted ANG II-mediated renal inflammation and fibrosis. Indeed, Sp1 is required for ANG II–induced fibrotic and inflammatory response [28], [29]. It is also reported that Sp1 can interact with Smad3 to enhance TGF-β-induced fibrotic response [29]–[31]. Thus, ANG II-induced up-regulation of Sp1 expression and activation of Smad3 may cooperate in the development of ANG II-induced renal fibrosis as seen in WT mice. Whereas, deletion of Smad7 may result in a further increase in Sp1-Smad3 interaction, thereby enhancing ANG II-mediated renal fibrosis. In addition, Sp1 is capable of interacting with NF-κB to play a critical role in autoimmune disease and cancer [32], [38]. Thus, enhanced both ANG II-induced up-regulation of Sp1 expression and activation of NF-κB signaling may account for exacerbation of ANG II-induced renal inflammation in Smad7 KO mice.

Furthermore, loss of miR-29 may also be a mechanism through which disruption of Smad7 enhances Ang II-mediated renal fibrosis and inflammation. miR-29 exerts an anti-fibrotic function through direct targeting of the 3'UTR regions in the mRNA for collagens I, III and IV and fibrillin and elastin [39]. We have previously demonstrated that Smad3 can physically interact with the miR-29b promoter to negatively regulate miR-29b expression in response to TGF-β1 in vitro and in obstructive nephropathy [27]. miR-29 is also negatively regulated by the NF-κB-YY1-miR-29 regulatory circuit in cancer cells [33]. Thus, down-regulation of miR-29 in ANG II-mediated hypertensive nephropathy may be attributed to activation of both TGF-β/Smad3 and NF-κB pathways. Moreover, miR-29 can inhibit TGF-β1 and Sp1 expression [40], [41]. Taken together, ANG II-induced loss of miR-29b via the TGF-β/Smad3 and NF-κB-dependent mechanism may result in further increase in TGF-β1 and Sp1-dependent renal injury. Therefore, loss of miR-29 may also be a new mechanism by which deletion of Smad7 enhanced ANG II-mediated renal injury via the Sp1-TGF-β/Smad3-NF-κB-miR29 auto-regulatory loop.

It should be pointed out that although Smad7 exerts its protective role in both renal fibrosis and inflammation, role of TGF-β1 under disease conditions is highly diverse due to the complexity of its downstream signaling pathway [42]. In the context of fibrosis and inflammation, Smad3 is pathogenic, while Smad2 and Smad7 are protective. Smad4 exerts its diverse roles by transcriptionally enhancing Smad3-mediated renal fibrosis while inhibiting NF-κB-driven renal inflammation [42]. In addition, unlike active TGF-β1 which mediates progressive renal fibrosis [43], mice overexpressing latent TGF-β1 are protected against progressive renal inflammation and fibrosis in obstructive and immunologically-induced crescentic glomerulonephritis via the Smad7-dependent mechanism [35], [44], [45]. All these studies suggest that specific targeting the downstream TGF-β/Smad3 signaling pathway by overexpression of Smad7, rather than blocking the general effect of TGF-β1 with neutralizing antibodies, may represent a specific and effective therapeutic strategy for kidney disease [46].

In summary, the current study found that disruption of Smad7 significantly increases Ang-II induced renal fibrosis and inflammation. Enhanced Sp1-TGF-β1/Smad3-NF-κB signaling may be the major mechanistic pathway by which deletion of Smad7 promotes ANG II-mediated renal fibrosis and inflammation. Thus, Smad7 plays a protective role in ANG II-induced hypertensive nephropathy. Results from this study suggest that strategies that can increase Smad7 levels may have therapeutic potential for hypertensive nephropathy.
